# AGPAT3 Regulates Immune Microenvironment in Osteosarcoma via Lysophosphatidic Acid Metabolism

**DOI:** 10.32604/or.2025.070558

**Published:** 2025-12-30

**Authors:** Shenghui Su, Yu Zeng, Jiaxin Chen, Xieping Dong

**Affiliations:** JXHC Key Laboratory of Digital Orthopaedics, Jiangxi Provincial People’s Hospital, The First Affiliated Hospital of Nanchang Medical College, Nanchang, 330006, China

**Keywords:** 1-acylglycerol-3-phosphate O-acyltransferase 3, osteosarcoma, single-cell, tumor-associated macrophages, lysophosphatidic acid

## Abstract

**Background:**

Recent studies have shown glycerolipid metabolism played an essential role in multiple tumors, however, its function in osteosarcoma is unclear. This study aimed to explore the role of glycerolipid metabolism in osteosarcoma.

**Methods:**

We conducted bioinformatics analysis using data from the Therapeutically Applicable Research to Generate Effective Treatments (TARGET) database and single-cell RNA sequencing. Least Absolute Shrinkage and Selection Operator (LASSO) regression was used to identify the Glycerolipid metabolism-related genes associated with the clinical outcome of osteosarcoma. Tumor-associated macrophages (TAMs) and their interactions with immune cells were examined through single-cell analysis and co-culture experiments. Virtual screening was employed to identify the potential lysophosphatidic acid receptor 6 (LPAR6) inhibitors.

**Results:**

Glycerolipid metabolism-related genes 1-acylglycerol-3-phosphate O-acyltransferase 3 (*AGPAT3*) and aldehyde dehydrogenase 7 family member A1 (*ALDH7A1*) were identified as key prognostic genes in osteosarcoma, with high *AGPAT3* expression correlating with improved survival. Single-cell analysis revealed that *AGPAT3* expression is associated with tumor immune microenvironment, particularly with TAMs. Knockdown of *AGPAT3* in osteosarcoma cells resulted in elevated lysophosphatidic acid (LPA) levels, which regulated the immune environment, inhibiting cytotoxic T cell function through TAMs’ LPAR6 signaling. LPAR6 signaling in TAMs mediates immune regulation through cytokine secretion, including interleukin-6 (IL-6) and interleukin-10 (IL-10). Further drug virtual screening identified Dutasteride as a potential inhibitor of LPAR6.

**Conclusion:**

*AGPAT3* is an important gene related to the prognosis of osteosarcoma. Its ability to modulate LPA signaling and TAM activity offers promising therapeutic opportunities for improving osteosarcoma treatment, particularly in immunotherapy contexts.

## 1 Introduction

Osteosarcoma is a malignant bone tumor with a complex etiology that threatens the health of children and adolescents. Osteosarcoma is characterized by a high rate of disability and a tendency for metastasis, with a particularly poor prognosis for metastatic patients [1,2]. The most common sites of metastasis include the lung and lymph node. Patients with distant metastasis have a worse prognosis compared to those with hematogenous metastasis, with a survival rate of only 10.9% [3–5]. The combination of neoadjuvant chemotherapy and surgical intervention has remarkably improved the overall survival rate for osteosarcoma patients to 60%–70% [5,6]. However, osteosarcoma treatment has stagnated over the past three decades, with 10%–40% patients experiencing recurrence or distant metastasis during therapy, resulting in poor clinical outcomes [7]. Therefore, a thorough investigation of the molecular mechanisms underlying metastasis in osteosarcoma is crucial for improving patient prognosis.

Recent studies have highlighted the essential role of glycerolipid metabolism in various cancers, including breast, ovarian, and prostate cancers [8,9]. Dysregulation of glycerolipid metabolism has been identified as a contributor to tumor proliferation and metastatic progression by providing essential fatty acids and energy substrates [10]. Evidence demonstrates that glycerolipid metabolism-related signatures (GLMS) are associated with overall survival in colon cancer. Patients with lower glycerolipid metabolism-related gene expression exhibit poorer clinical outcomes and increased immune evasion, demonstrating the essential role of lipid metabolism in the tumor microenvironment [11]. In ovarian cancer, lysophosphatidic acid (LPA) produced by phospholipase A2 has been confirmed to facilitate the proliferation and invasion of tumor cells. Previous reports have demonstrated that the phosphatidic acid acyltransferase 1-acylglycerol-3-phosphate O-acyltransferase 3 (AGPAT3) regulates lipid metabolism and ferroptosis, which could influence the survival of tumor cells [12–14].

Furthermore, the interaction between tumor cells and the immune microenvironment significantly influences lipid metabolism [15,16]. Cancer-associated adipocytes release fatty acids and pro-inflammatory mediators that support tumor growth and metastasis [17]. Glycerolipid metabolism is associated with tumor biology across various cancers. Glycerolipid metabolism plays an essential role in clinical outcomes, metastatic potential, and interactions within the tumor microenvironment. However, the role of glycerolipid metabolism and the function of related essential genes in osteosarcoma progression were still unclear. Therefore, this understanding could pave the way for novel prognostic markers and treatment strategies in cancers such as osteosarcoma. We hypothesized that the glycerolipid metabolism and related metabolites influenced the progression of osteosarcoma. To explore this, we examined the gene expression in glycerolipid metabolism and analysed its correlation with clinical outcome. To further illustrate its role in immune microenvironment, single-cell analysis was used to explore the changes in immune cells. Following the bioinformatic analysis, co-culture and flow cytometry assays were conducted to explore the impact of essential genes in immune cells.

## 2 Materials and Methods

### 2.1 Data Source

The RNA sequencing data and corresponding clinical data of osteosarcoma patients were acquired in the Therapeutically Applicable Research to Generate Effective Treatments (TARGET. https://www.cancer.gov/ccg/research/genome-sequencing/target (accessed on 04 September 2025)) project. The prognosis and RNA sequencing (RNA-seq) data were used for clustering and survival analysis. The osteosarcoma single-cell RNA sequencing (scRNA-seq) data obtained from Gene Expression Omnibus (GEO) Database (https://www.ncbi.nlm.nih.gov/geo/), with datasets record numbers GSE152048, GSE21257 and GSE162454. Genes associated with glycerolipid metabolism (KEGG_GLYCEROLIPID_METABOLISM) were acquired using the Molecular Signatures Database (MSigDB, https://www.gsea-msigdb.org/gsea/msigdb (accessed on 04 September 2025)). Single-sample gene set enrichment analysis (ssGSEA) and Spearman’s correlation andlysis was used for pathway analysis.

### 2.2 Consensus Clustering and Pathway Characteristics Analysis

We utilized RNA-seq and clinical data of osteosarcoma patients from TARGET and GEO, applying unsupervised clustering to classify patients into distinct molecular subtypes. The number of clusters and their stability were determined using the “ConsensusClusterPlus” (version 1.36.0) R package [18]. Optimal clustering was assessed using the consensus matrix, the distribution function (CDF), and the area under the CDF curve. Additionally, we performed functional enrichment analysis on gene expression profiles across different groups using Gene Ontology (GO) databases and “GSVA” (version 2.2.0) [19]. Immune-related signature scores were analysed using the “IOBR” package (version 2.0) and Cell-type Identification by Estimating Relative Subsets of RNA Transcripts (CIBERSORT) method were used for immune cells analysis [20,21]. Kaplan-Meier survival analysis was conducted to assess the prognosis of patients with various signatures.

### 2.3 Identification of the Candidate Genes

In our study, we employed the LASSO regression method and implemented it through the “glmnet” package (version 4) [22] to analyze our dataset. The selection of potential genes was guided by an optimal regularization parameter λ, which was ascertained using the 1-SE (standard error) rule. To quantify the risk, we constructed a scoring system that integrated the expression values of the selected genes with their associated LASSO coefficients.

### 2.4 Single-Cell RNA Sequencing Data Analysis

Single-cell RNA sequencing analysis was conducted using the R environment (version 4.2.3). Raw data underwent processing and quality control, with further analyses were carried out using the “Seurat” package (version 4.1.4) [23]. For quality control purposes, we retained genes that were expressed in a minimum of three cells. Cells exhibiting over 10% mitochondrial read counts or fewer than 5% ribosomal read counts were excluded from the analysis. Cells expressing fewer than 200 or more than 5000 genes were removed. Potential doublet cells were identified using the “DoubletFinder” tool (version 2.0.6) [24]. “Harmony” (version 1.2.3) [25] was employed to mitigate batch effects. Cell annotation was conducted manually according to the gene expression profiles and the reference database [26]. Cell communication analysis was conducted using “CellChat” (version 1.3.1). Heatmap, dot plot and chord diagram was used to illustrate the corresponding results [27]. The cell trajectories analysis of TAMs was conducted using the “Slingshot” (version 2.16.0) [28].

### 2.5 Cell Lines

The human osteosarcoma cell lines 143B (RRID: CVCL_2270), HEK293T (CVCL_0063), and THP-1 (RRID: CVCL_0006) were obtained from Pricella Life Science & Technology Co., Ltd. (Wuhan, China). The cells were cultured according to the instructions from the American Type Culture Collection (ATCC). In specific, HEK293T and 143B cells were cultured in DMEM medium (Gibco, Grand Island, NY, USA, #11965092) with 10% fetal bovine serum (Gibco, Auckland, New Zealand, #10099141C) and 1% Penicillin-Streptomycin (Gibco, Grand Island, NY, USA, #15140122), at 37°C and 5% CO_2_. The THP-1 cell was cultured in RPMI 1640 medium (Gibco, Grand Island, NY, USA, #11875093) with 10% fetal bovine serum (Gibco, Auckland, New Zealand, #10099141C), 0.05 mM 2-mercaptoethanol (Gibco, Grand Island, NY, USA, #21985023), and 1% Penicillin-Streptomycin (Gibco, Grand Island, NY, USA, #15140122), at 37°C and 5% CO_2_. The cells used were all passed short tandem repeat (STR) profiling and were all tested free of mycoplasma contamination before the experiments.

### 2.6 Plasmid Construction and Lentiviral Transduction

To knock down AGPAT3, a short hairpin RNA (shRNA) targeting *AGPAT3* was designed and cloned into the pLKO.1 vector (IGE Biotechnology, Guangzhou, China). The lentiviral packaging plasmids (Addgene, Watertown, MA, USA, #12251, #12253, and #12259) were transfected together with the AGPAT3 knockdown plasmid using Lipofectamine3000 (Invitrogen, Carlsbad, CA, USA, #L3000015). After transfecting HEK293T cells for 48 h, the corresponding lentiviral-containing supernatant was collected. The 143B cell was infected immediately using the fresh lentiviral-containing supernatant using polybrene (Sigma-Aldrich, St. Louis, MO, USA, #TR-1003). Then puromycin (Gibco, Grand Island, NY, USA, #A1113803) was used for selection for one week. Knockdown efficiency of AGPAT3 was analysed using Western blot.

### 2.7 Western Blot Analysis

The 143B and THP-1-derived macrophage proteins were isolated using RIPA buffer, supplemented with 1 mM phenylmethanesulfonyl fluoride (PMSF; Beyotime, Shanghai, China, #ST507-10 mL). The concentration of proteins was measured using the bicinchoninic acid (BCA) method (Beyotime, Shanghai, China, #P0011). A total of 30 μg of protein per sample was loaded onto each lane, using a 10% SDS-polyacrylamide gel for electrophoresis. After separation, proteins were transferred onto a 0.22 μm PVDF membrane (Millipore, St. Louis, MO, USA, # GVWP04700) using wet transfer at 120 V for 90 min in ice. The membrane was blocked with 5% non-fat dry milk in TBST (Tris-buffered saline with 0.1% Tween-20) for 1 h at room temperature. For blocking, the membrane was treated with a 5% solution of non-fat milk and then incubated with primary antibodies overnight at 4°C (AGPAT3:25723-1-AP, Proteintech, Wuhan, China, dilution 1:1000; IL-10: #12163, CST, Danvers, MA, USA, dilution 1:1000; IL-6: #12912, CST, dilution 1:1000; LPAR6: #PA5-67745, Invitrogen, dilution 1:1000; CD97: GTX637674, Gnetex, Irvine, CA, USA, dilution 1:1000). Subsequently, membranes were washed 3 times for 5 min each in TBST. Then the membranes were incubated with HRP-conjugated antibodies (goat anti-rabbit IgG, Jackson ImmunoResearch, 1:5000 dilution) at room temperature for 1 h. Protein bands were visualized using High-Sig ECL substrate (Tanon, Shanghai, China, #180-501) and imaged with a chemiluminescent imaging system (Tanon 5200).

### 2.8 Macrophage Stimulation and Co-Culture Assay

The THP-1 cells were used to differentiate macrophages. THP-1 cells were incubated with 150 nM phorbol 12-myristate 13-acetate (PMA; #P8139 Sigma-Aldrich) for a period of 24 h. For the co-culture analysis, THP-1 derived-macrophage were cultured in a 6-well cell culture and cocultured with the indicated 143B cells seeded in inserts (pore size 0.4 μm, Falcon, Durham NC, USA #353090). The co-culture was conducted for 48 h. Following this co-culture period, the levels of a specific cytokine were assessed using enzyme-linked immunosorbent assay (ELISA). For treatment, the THP-1 derived macrophage was treated with 9-xanthenylacetic acid (XAA) 200 nM for 12 h. The XAA were synthesized in collaboration with a chemical synthesis company as previously described [29].

### 2.9 ELISA-Based Assay

To detect the levels of LPA, supernatants were collected, and ELISA analysis was conducted using kits (Echelon Biosciences, Salt Lake City, UT, USA, K-2800S) following the protocols. After the macrophage and 143B cell co-culture experiment for 12 h, the supernatants were collected. ELISA kits (IL-6: ab178013, Abcam, Waltham, MA, USA; IL-10: D1000B, R&D Systems, Minneapolis, MN, USA) were used to detect the cytokine IL-6 or IL-10 levels, refer per the corresponding guidelines.

### 2.10 Virtual Screening of LPAR6 Inhibitors

The docking study based on structure was performed using the Autodock Vina (version 2.0) program, with the three-dimensional structure of the LPAR6 protein obtained from previous studies [30]. The structures of Food and Drug Administration (FDA) approved drugs were downloaded from ZINC20 database (https://zinc.docking.org/) [31]. FDA-approved drugs were converted into 3D models using Open Babel (version 3.1.1). Docking was conducted to explore the potential conformations and orientations of ligands at the binding site. All bonds of the ligands were set as rotatable. Protein-ligand-flexible docking calculations were conducted according to the Lamarckian Genetic Algorithm (LGA) method [32]. The binding sites of key residues in LPAR6 were identified as the binding site of lysophosphatidic acid. The best confirmation was determined based on binding energy. The Dutasteride (2 μM, 12 h) was used for co-culture treatment.

### 2.11 Statistical Analysis

All statistical analyses and graphical visualizations were conducted using R v4.2.3 (http://www.r-project.org) and GraphPad (version 8.0.2, Boston, MA, USA). All the experiments were conducted with 3 replicates, and the data presented are representative of a single experiment. We performed statistical analyses using Student’s *t*-test and One-way ANOVA. Overall survival was assessed using the Kaplan-Meier method, using the log-rank test. Differences with *p*-values less than 0.05 were considered statistically significant.

## 3 Results

### 3.1 Glycerolipid Metabolism-Based Molecular Clusters in the Patient Cohort and Characteristics

**Figure 1:**
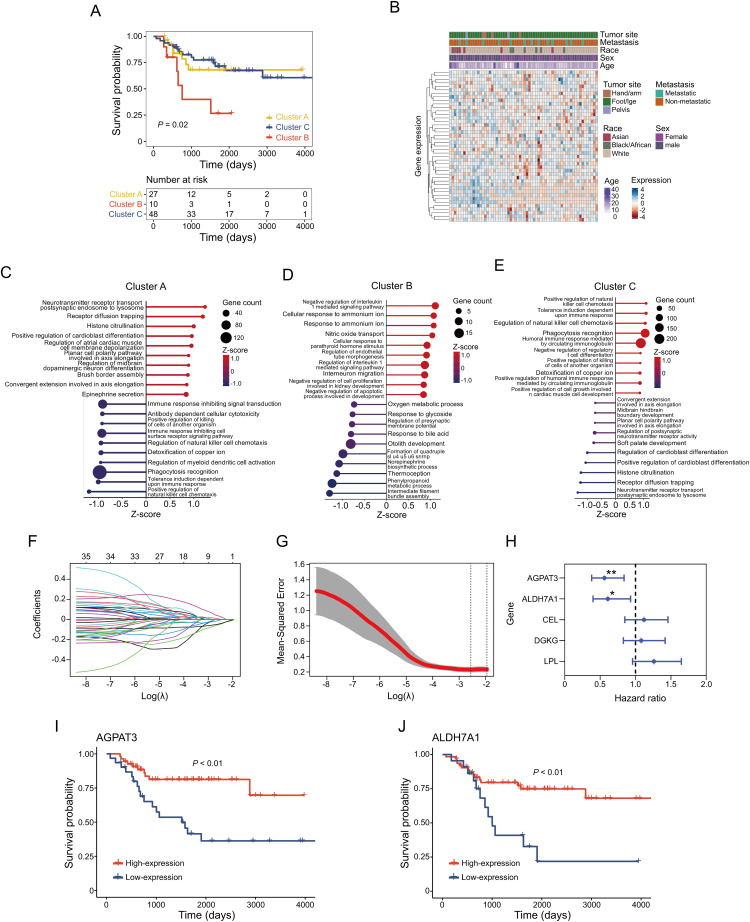
Glycerolipid metabolism-based clustering in osteosarcoma cohort and corresponding signatures: (**A**) Kaplan–Meier survival analysis of the three clusters. (**B**) Correlation between expression of glycerolipid metabolism gene and clinical characteristics. (**C–E**) Top 10 upregulated and downregulated pathways in different clusters. (**F**) Coefficient of glycerolipid metabolism-based genes. (**G**) Identification of the parameter (lambda) in LASSO. (**H**) Cox analysis of identified 5 genes in regression. Kaplan-Meier survival analysis of *AGPAT3* (**I**) and *ALDH7A1* (**J**). *p < 0.05, **p < 0.01, by log-rank test (**H**)

To explore the relationship between glycerolipid metabolism and osteosarcoma clinical outcome, we conducted consensus clustering on TARGET osteosarcoma cases using 49 genes from the KEGG glycerolipid metabolism pathway. Based on minimal overlap within the consensus matrix and consistency in the cumulative distribution function (CDF) curve trend, the samples were divided into three distinct clusters (Fig. 1A). Survival analysis across these clusters showed significant differences in prognosis, with patients in Cluster B demonstrating worse outcomes compared to the Clusters A or C (*p* = 0.02) (Fig. 1A). Furthermore, we assessed the association between the clinical features such as sex, age, metastatic status and glycerolipid metabolism gene expression. Results showed there was no significant correlation between gene expression and these clinical characteristics (Fig. 1B). To further illustrate the characteristics of those clusters, gene set variation analysis (GSVA) was used. In Cluster A, we identified alterations in pathways involving NK cell regulation, myeloid cell activation, and immune response. (Fig. 1C). Cluster B, associated with poorer prognosis, exhibited significant changes in metabolic pathways, including Oxygen metabolic process and Phenylpropanoid metabolic process (Fig. 1D). In Cluster C, patients with better clinical outcome, there were notable changes in immune-related pathways such as NK cell activation and T cell differentiation (Fig. 1E).

In order to explore the prognosis-related genes in the indicated metabolism pathway, we performed LASSO-penalized Cox regression on the TARGET osteosarcoma cohort. This analysis identified five potential prognostic genes related to glycerolipid metabolism (*AGPAT3*, *ALDH7A1*, *CEL*, *DGKG*, and *LPL*) (Fig. 1F–H). Using the minimum criterion for penalty parameter selection, *AGPAT3* showed the lowest hazard ratio at 0.56 (*p* < 0.01) (Fig. 1H). Survival analysis of genes with *p* < 0.05 in the Cox regression confirmed that high expression of *AGPAT3* and *ALDH7A1* positively influenced patient prognosis, with high-expression groups exhibiting better outcomes (Fig. 1I,J). These findings suggest that glycerolipid metabolism potentially affected the tumor immune microenvironment. Genes *AGPAT3* and *ALDH7A1* in the glycerolipid metabolic pathway may serve as prognostic markers and play key roles in osteosarcoma.

### 3.2 Immune Signatures of AGPAT3-Based Osteosarcoma Cohort

**Figure 2:**
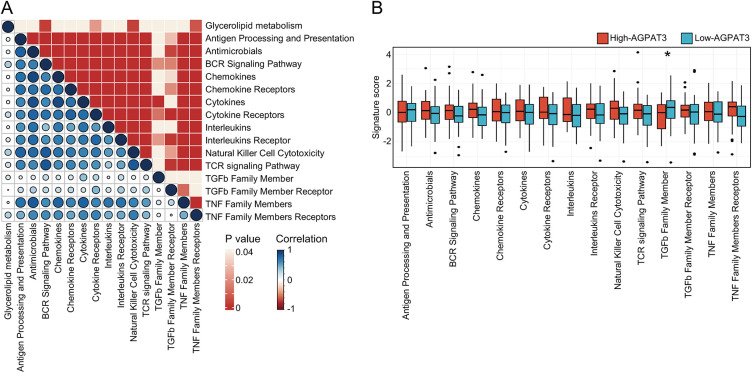

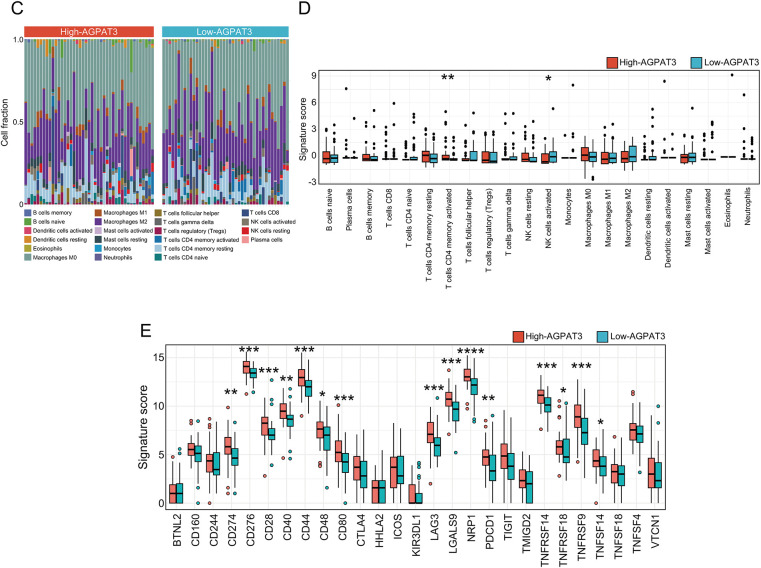
Immune signatures of AGPAT3-related groups: (**A**) The correlation analysis between the signature score of glycerolipid metabolism and immune-related pathways, correlation value shown in left and *p*-value shown in right. (**B**) Estimated scores and differential expression analysis of immune-related pathways in high- and low-*AGPAT3* expression groups. (**C**) Immune cell analysis in high- and low-*AGPAT3* expression groups. (**D**) Signature score and differential expression analysis between high- and low-*AGPAT3* expression groups. (**E**) Expression of immune checkpoint genes in high- and low-*AGPAT3* expression groups. Data are presented as the mean ± SD. *p < 0.05, **p < 0.01, ***p < 0.001, ****p < 0.0001, by Student *t*-test in (**B**,**D**,**E**)

Previous consensus clustering and survival analysis suggested glycerolipid metabolism was associated with the regulation of the immune microenvironment in osteosarcoma. To further explore the role of essential prognostic genes of glycerolipid metabolism in osteosarcoma immune microenvironment, we grouped the cohort based on the expression of *AGPAT3*. Further single-sample gene set enrichment analysis (ssGSEA) was used to investigate the glycerolipid metabolism pathway scores and other 15 immune-related pathway scores for each sample, followed by a correlation analysis. Results revealed the glycerolipid metabolism was significantly associated with cytokines receptors, B cell receptor signaling pathway and many other immune-related pathways (Fig. 2A). The different expression analysis of immune pathway scores between the high- and low-*AGPAT3* expression groups demonstrated a significant diversity in the TGF-β pathway (*p* < 0.05) (Fig. 2B). We also performed a validation in GSE21257 cohort and results demonstrated *AGPAT3* was associated with multiple immune markers (Supplementary Fig. S1).

Using the CIBERSORT method, we analyzed the immune cell composition of each sample to investigate the immune cell signature of osteosarcoma samples from TARGET database (Fig. 2C). The analysis showed elevated levels of CD4 memory T cells in the high-*AGPAT3* group, while activated NK cells were more abundant in the low-*AGPAT3* group (Fig. 2D). Moreover, the related immune checkpoint characteristics demonstrated significant differences between many immune-related genes, including *CD274, CD276, CD28, CD40, CD44, CD48, CD80, LAG3, LGALS9, NRP1, PDCD1, TNFRSF14, TNFRSF18, TNFRSF9* and *TNFSF14*, which were highly expressed in high-AGPAT3 group. (Fig. 2E). These results indicate that glycerolipid metabolism is closely associated with immune regulation in osteosarcoma.

### 3.3 Tumor Microenvironment Profiling with scRNA-Seq Dataset

**Figure 3:**
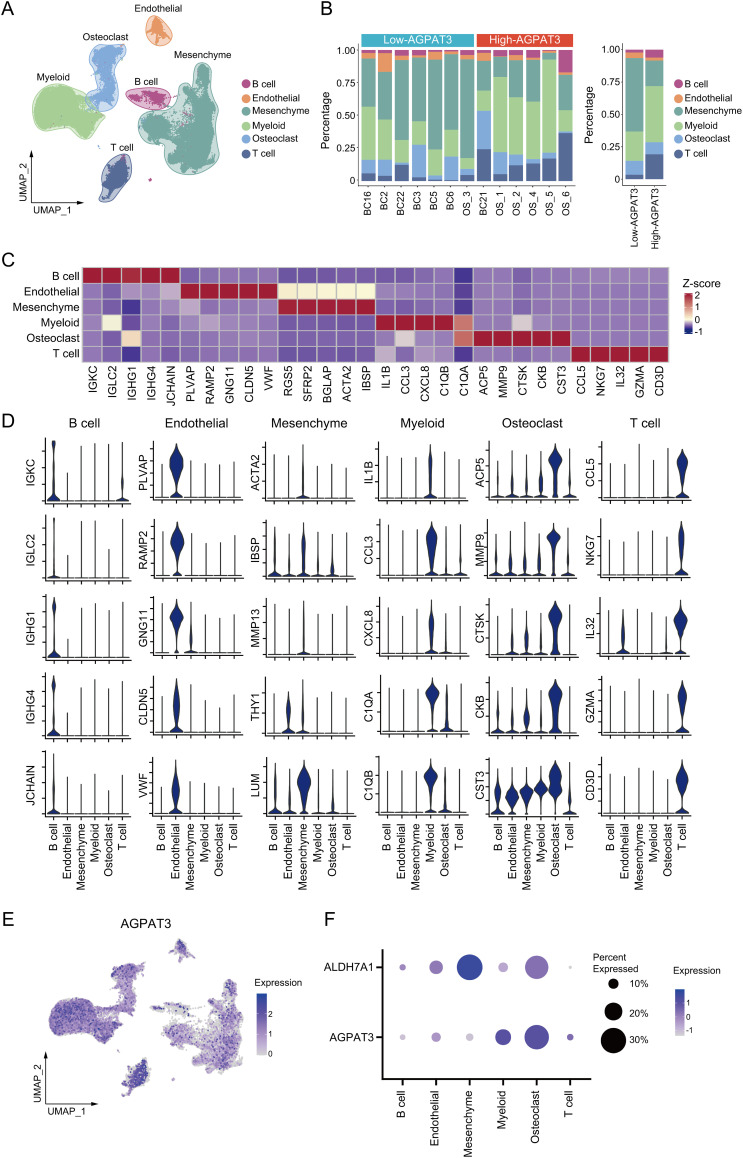
Single-cell analysis of osteosarcoma sample: (**A**) Cell clustering of osteosarcoma samples from GSE152048 and GSE162454. (**B**) Specific cell proportions of different samples or different *AGPAT3* expression groups. (**C**,**D**) Expression of marker genes in indicated cell types. (**E**) Expression of *AGPAT3* in different cells. (**F**) Expression of *AGPAT3* and *ALDH7A1* in different cells

To illustrate the function of AGPAT3 in the osteosarcoma immune microenvironment, we used single-cell analysis. The osteosarcoma scRNA-seq dataset GSE152048 and GSE162454, containing primary osteosarcoma tissues, were used for further analysis. After clustering analysis, all the cells were divided into different lineages: T cells (marker genes: *CCL5, NKG7, IL32, GZMA, CD3D*), mesenchymal cells (marker genes: ACTA2, IBSP, MMP13, THY1, LUM), B cells (marker genes: *IGKC, IGLC2, IGHG1, IGHG4, JCHAIN*), myeloid cells (marker genes: *IL1B, CCL3, CXCL8, C1QA, C1QB*), osteoblasts (marker genes: *ACP5, MMP9, CTSK, CKB, CST3*), and endothelial cells (marker genes: *IL1B, CCL3, CXCL8, C1QA, C1QB*) (Fig. 3A). Based on *AGPAT3* expression levels, samples were divided into high- or low-*AGPAT3* expression groups, each displaying distinct proportions of cell types. Notably, there are more myeloid cells and fewer mesenchymal cells in high-AGPAT3 expression samples. (Fig. 3B). Each cell cluster demonstrated unique gene expression profiles (Fig. 3C,D). *AGPAT3* was predominantly expressed in myeloid cells and osteoblasts, while *ALDH7A1* was expressed most in mesenchymal cells (Fig. 3E,F).

**Figure 4:**
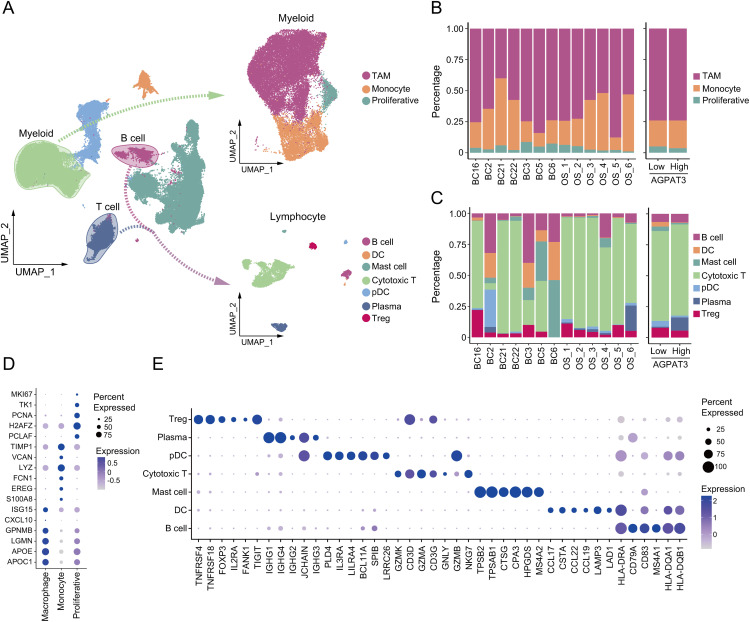
Cluster of myeloid cells and lymphocytes in osteosarcoma: (**A**) Detailed cluster of myeloid cells and lymphocytes in osteosarcoma samples. (**B**) Cell proportion of myeloid cells in different samples and *AGPAT3* expression group. (**C**) Cell proportions of B and T cells in different samples and the *AGPAT3* expression group. (**D**) Expression of the marker gene of myeloid cells. (**E**) Expression of marker genes of lymphocytes

To further investigate the difference in immune cells, we grouped and clustered the myeloid and lymphocyte cells. Myeloid cells were classified into TAMs (marker genes: *ISG15, CXCL10, GPNMB, LGMN, APOE, APOC1*), monocytes (marker genes: *TIMP1, VCAN, LYZ, FCN1, EREG, S100A8*), and proliferative cells (marker genes: *MKI67, TK1, PCNA, H2AFZ, PCLAM*). lymphocyte were clustered into different subtypes, including: mast cells (marker genes: *TPSB2, TPSAB1, CTSG, CPA3, HPGDS, MS4A2*), regulatory T cells (T-regs, marker genes: *TNFRSF4, TNFRSF18, FOXP3, TIGIT*), cytotoxic T cells (marker genes: *GZMK, CD3D, GZMA, CD3G, GNLY, GZMB*), B cells (marker genes: *HLA-DRA, CD79A, CD83, MS4A1, HLA-DQA1, HLA-DQB1*), plasma cells (marker genes: *IGHG1, IGHG4, IGHG2, JCHAIN, IGHG3, GZMB*), dendritic cells (DCs, marker genes: *CCL17, CSTA, CCL22, CCL19, LAMP3, LAD1*) and plasmacytoid dendritic cells (pDCs, marker genes: *PLD4, IL3RA, LILRA4, BCL11A, SPIB, LRRC26*) (Fig. 4A). Although there was no significant difference in the proportion of myeloid cells between the high- and low-*AGPAT3* expression groups (Fig. 4B), but the plasma cell abundance was significantly lower in the low-AGPAT3 group compared to the high-*AGPAT3* group (Fig. 4C). There were specific gene expression for each sub-cluster (Fig. 4D,E).

### 3.4 Cell Communication between TAMs and Lymphocytes in Osteosarcoma

**Figure 5:**
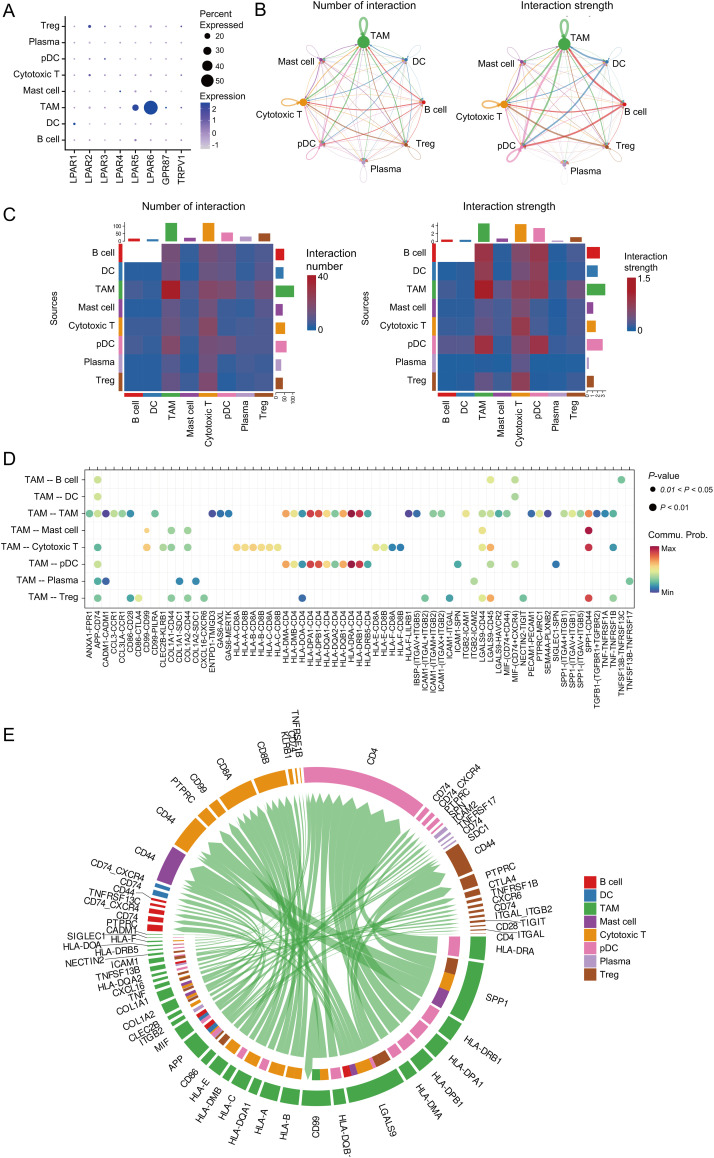
Cell-cell interactions between TAMs and other expression: (**A**) Expression of LPA receptors in immune cells. (**B**) Cell communication network of different cells, number of interactions (left); strength of interactions (right). (**C**) Heatmap showing the cell communication between different cells, number of interactions (left); strength of interactions (right). (**D**) The dot plot shows the cell communication between TAMs and other cells. (**E**) Chord diagram showing multiple cell interactions between TAMs and other cells

Analysis of the osteosarcoma immune microenvironment suggests that AGPAT3 may not regulate the immune microenvironment by influencing the number or proportion of immune cells. Instead, AGPAT3 may regulate the immune microenvironment through cell interaction. As a member of the AGPAT family, AGPAT3 can convert LPA into phosphatidic acid. LPA plays an essential role in cell signaling through lysophosphatidic acid receptor, participating in various biological processes, including MAPK and Rho signaling pathways, as well as cell proliferation [33]. To further explore the role of AGPAT3 and LPA signaling, we first analyzed the expression of LPAR across different cell types. Results showed *LPAR6* was the most highly expressed LPA receptor, mainly expressed in TAMs (Fig. 5A). Next, we used “CellChat” to explore ligand-receptor interactions among immune cells. The analysis demonstrated frequent and strong interactions between TAMs and other cell types. Moreover, cytotoxic T cells received the highest number of signals. The interactions of TAMs and cytotoxic T cells may play an essential role in the tumor immune evasion (Fig. 5B,C). Detailed analysis of TAM interactions with other cells (Fig. 5D) showed significant communication of TAMs or cytotoxic T cells (Fig. 5E). These cell communication results showed that low *AGPAT3* expression in osteosarcoma may lead to elevated LPA levels. The increased LPA may regulate TAM’s function and interaction with other cells through LPAR.

### 3.5 AGPAT3 Associated with TAMs-Induced Immunosuppressive Microenvironment

**Figure 6:**
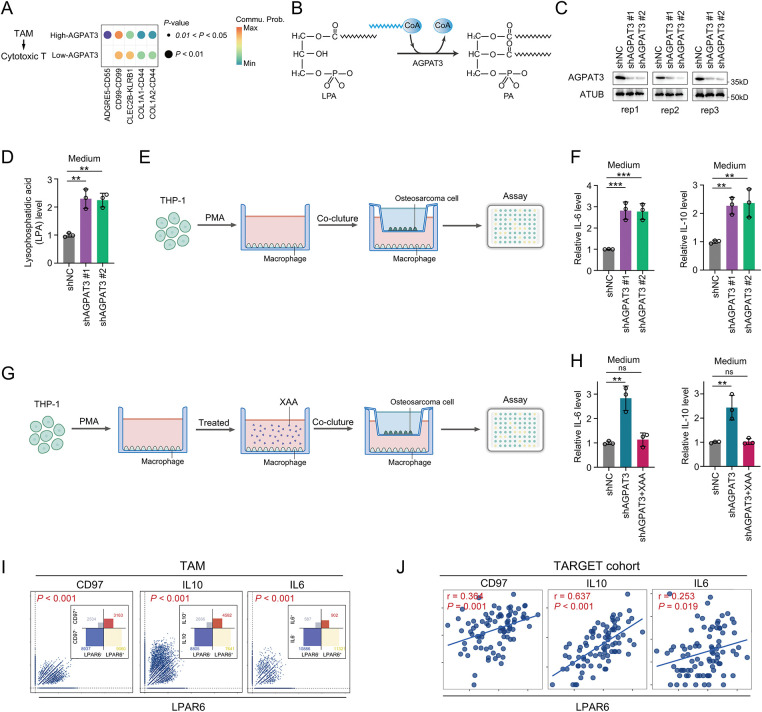

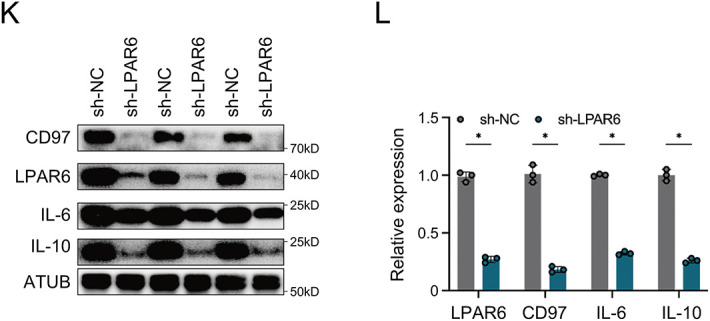
AGPAT3-mediated LPA metabolism influenced TAMs cytokine secretion: (**A**) Differential analysis of cell interactions between TAMs and cytotoxic T cells in different AGPAT3 expression groups. (**B**) A diagram showing AGPAT3 converts lysophosphatidic acid into phosphatidic acid. (**C**) Knockdown of AGPAT3 in 143B osteosarcoma cell lines. (**D**) Medium LPA level detected using ELISA (n = 3). (**E**) Co-culture experiment using *AGPAT3*-KD 143B cells and THP-1 derived. (**F**) Expression of IL-6 (left) and IL-10 (right) secreted by TAMs in a co-cultured experiment, detected by ELISA (n = 3). (**G**) Co-culture of *AGPAT3*-KD 143B cells with macrophages derived from THP-1 treated with XAA. (**H**) Level of IL-6 (left) and IL-10 (right) detected by ELISA (n = 3). (**I**) Correlation analysis of *LPAR6*, *CD97*, *IL10*, and *IL6* expression in TAMs. (**J**) Correlation analysis of *LPAR6*, *CD97*, *IL10*, and *IL6* expression in osteosarcoma cohort from TARGET. (**K**,**L**) Western blot analysis and corresponding quantification showing that *LPAR6* knockdown in THP-1-derived macrophages co-cultured with AGPAT3-silenced 143B cells reduces the expression of CD97, IL-10, and IL-6 (n = 3). Data are presented as the mean ± SD, ns not significant, *p < 0.05, **p < 0.01, and ***p < 0.001, by One-way ANOVA test (**D**,**F**,**H**), by Pearson correlation test (**I**,**J**)

To further illustrate the role of *AGPAT3* expression in cell-cell interaction, we further compared the cell communication in different AGPAT3 groups. There were different signals from TAMs to cytotoxic T cells (Fig. 6A). The ADGRE5-CD55 interaction exhibited significant changes, suggesting that *AGPAT3* expression may influence macrophage-cytotoxic T cell interaction (Fig. 6A). AGPAT3 converse LPA into phosphatidic acid (Fig. 6B). To explore whether AGPAT3 and LPA influence TAM functionality, we knocked down *AGPAT3* (AGPAT3-KD) in the osteosarcoma cell line 143B. LPA levels in the culture medium significantly increased following *AGPAT3* knockdown (Fig. 6C,D). Subsequently, we co-cultured THP-1-derived macrophages with 143B cells and analyzed cytokine secretion by TAMs (Fig. 6E). The regulatory cytokines IL-10 and IL-6 were significantly upregulated in TAMs induced by *AGPAT3* knockdown (Fig. 6F).

To further examine whether LPA synthesis mediated by osteosarcoma-derived AGPAT3 induces cytokine secretion through LPAR6, we treated THP-1-derived macrophages with XAA, an LPAR6 inhibitor [34], followed by co-culture experiments. Analysis showed that XAA significantly suppressed the AGPAT3-induced IL-10 and IL-6 upregulation in TAMs (Fig. 6G,H). Next, we investigated the correlation between *LPAR6*, *ADGRE5*, *IL10*, and *IL6* in scRNA-seq and the TARGET osteosarcoma cohort. In TAMs, *LPAR6* expression was positively correlated with *ADGRE5*, *IL10*, and *IL6* (Fig. 6I), which was further validated in the osteosarcoma cohort (Fig. 6J). Furthermore, western blot analysis showed that knockdown of LPAR6 in THP-1-derived macrophages co-cultured with *AGPAT3*-KD 143B cells led to a decrease in the expression levels of CD97, IL-10, and IL-6 (Fig. 6K,L).

These findings suggest that AGPAT3 may promote the immunosuppressive microenvironment formation and osteosarcoma immune evasion by regulating the ADGRE5-CD55 interaction between TAMs and T cells, by regulating TAM function through LPA-mediated.

### 3.6 Structure-Based Virtual Screen Identified Potential Inhibitors of LPAR6

**Figure 7:**
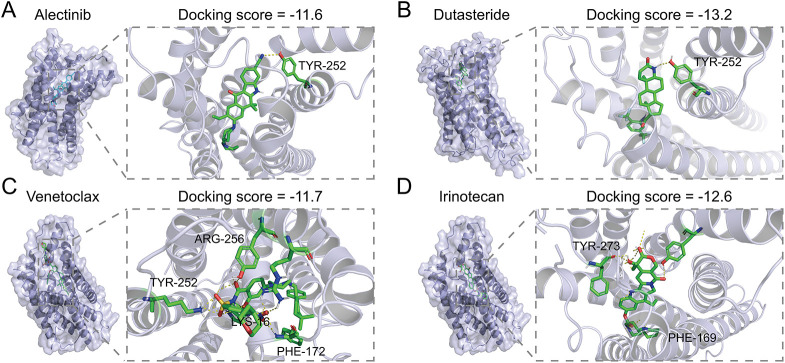

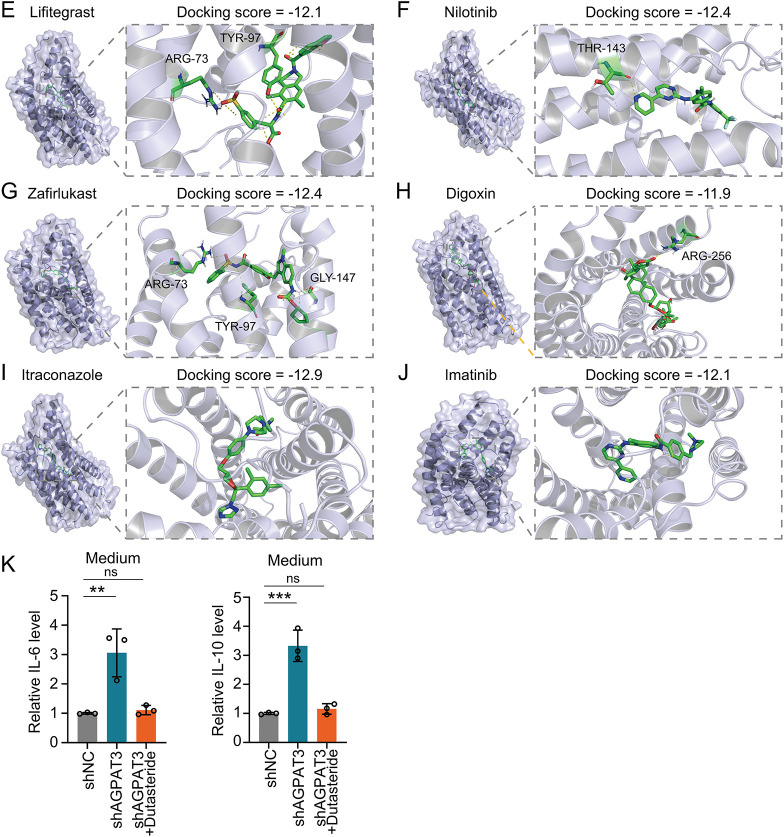
Structure-based virtual screen of LPAR6 using FDA-approved drug: the molecular docking of Alectinib (**A**), Dutasteride (**B**), Venetoclax (**C**), Irinotecan (**D**), Lifitegrast (**E**), Nilotinib (**F**), Zafirlukast (**G**), Digoxin (**H**), Itraconazole (**I**), Imatinib (**J**) with LPAR6. (**K**) Level of IL-6 (left) and IL-10 (right) detected by ELISA in co-culture model with Dutasteride (2 μM) (n = 3). Data are presented as the mean ± SD. ns, not significant, **p < 0.01, and ***p < 0.001, by One-way ANOVA test in (**K**)

To further explore the potential therapeutic value of LPAR6, we further conducted structure-based virtual screening using FDA-approved drugs to find inhibitors that might block the function of LPAR6. Using the ZINC20 database [31], we retrieved the FDA-approved drugs and converted their structures into 3D models. We obtained the structure of LPAR6 from previous studies [30]. Next, we used AutoDock Vina to dock the drugs with LPAR6. The top 10 drugs with the best (lowest) docking scores were selected. The docking results were visualized with yellow dashed lines indicating hydrogen bonds, and the interacting amino acid residues were labeled accordingly (Fig. 7A**–**J). Further co-culture experiments showed that Dutasteride could suppress the LPA-induced IL-10 and IL-6 upregulation in TAMs (Fig. 7K). Among these drugs, Dutasteride demonstrated the best docking scores. In summary, Dutasteride likely binds to and inhibits LPAR6, suggesting its potential as an inhibitor of the LPA signaling pathway.

## 4 Discussion

Cumulative evidence indicates that phosphatidic acid (PA) and LPA are dysregulated in various cancers and play critical roles in tumor progression [35–37]. As key signaling molecules, PA and LPA regulate processes such as apoptosis, proliferation, migration, senescence, and the tumor immune microenvironment [38]. In ovarian cancer, LPA is enriched in the immunosuppressive tumor microenvironment, where it promotes dendritic cell-derived EP4 signaling, suppressing type I IFN signaling. Inhibiting LPA production has been shown to enhance the efficacy of immunotherapy [39]. In breast cancer, the long non-coding RNA SNHG9 interacts with PA and LATS1, facilitating phase separation of LATS1 and modulating the YAP signaling pathway, thereby influencing malignant progression [40]. Research on osteosarcoma has primarily focused on the effects of LPA and PA on osteosarcoma cells themselves [41,42]. Some studies have reported the association of *LPAR5*^*+*^ macrophages with osteosarcoma prognosis [43], but comprehensive analyses of key molecules in the glycerolipid metabolic pathway remain limited. Our study identified prognostic features related to glycerolipid metabolism in osteosarcoma and highlighted key genes associated with clinical outcomes. We found that low expression of *AGPAT3* and *ALDH7A1* is significantly correlated with poor prognosis in osteosarcoma patients. *ALDH7A1* is a conserved member of the aldehyde dehydrogenase family, catalyzing the oxidation of aldehyde [44]. Recent studies have shown that ALDH7A1 is responsible for the NADH generation in the cell membrane, which could regulate the ferroptosis in tumor cells [45]. Moreover, ALDH7A1 was reported to promote tumor progression in both lung carcinoma and pancreatic ductal adenocarcinoma [46,47]. In this research, high expression of *AGPAT3* or *ALDH7A1* indicated better clinical outcomes. In osteosarcoma, the combination of *AGPAT3* or *ALDH7A1* may be used to predict the prognosis. However, further research is required to illustrate the function of ALDH7A1 in osteosarcoma.

Immunotherapy has proven to be a cornerstone in the treatment of various cancers, and increasing evidence highlights the critical roles of PA and LPA in regulating the tumor immune suppressive microenvironment [39,48]. LPA plays an essential role in cell signaling through lysophosphatidic acid receptor, participating in various biological processes, including MAPK and Rho signaling pathways, as well as cell proliferation [33]. Moreover, previous reports have shown that LPA-LPAR could induce the IL-6 and IL-10 expression and secretion broadly [49–51]. In melanoma, LPA modulates oxidative phosphorylation reprogramming in CD8^+^ T cells through the LPAR signaling pathway, promoting T cell exhaustion and immune suppression, ultimately fostering an immune-tolerant state [52]. In KRAS-TP53 mutant non-small cell lung cancer, autotaxin and LPA are markedly upregulated in tumors resistant to anti-PD-1 therapy, where they suppress CD8^+^ T cell infiltration via LPAR5 signaling [53]. This study uncovers the significant connection between the glycerolipid metabolic pathway and immune-related pathways in osteosarcoma, such as NK cell activation. Specifically, *AGPAT3*, which mediates the conversion of LPA to PA, correlates with immune cell composition. In clinical practice, there were trials focusing on the diagnostic efficiency of LPA in ovarian cancer early detection [54]. Moreover, the LPAR antagonists, BMS-986020, BMS-986278, and SAR-100842, were also enrolled in therapeutic clinical trials for pulmonary and autoimmune disease [55–57]. These clinical studies have provided novel insights into the role of AGPAT3 in the osteosarcoma immune microenvironment and identified it as a potential target to enhance immunotherapy outcomes.

TAMs are pivotal regulators of immune homeostasis in the TME [58,59]. They are abundant in the TME and play key roles in extracellular matrix remodeling, metastasis, immune suppression, chemoresistance, and resistance to immune checkpoint blockade therapies [60–64]. In breast cancer, FOLR2^+^ macrophages interact with CD8^+^ T cells, effectively activating them, and the density of FOLR2^+^ macrophages is strongly associated with patient survival [65]. In glioblastoma, cholesterol accumulation within TAMs supplies cholesterol-rich phospholipid fragments to tumor cells, fulfilling the high metabolic demands of mesenchymal glioblastoma and driving malignant progression [66]. Previous reports have shown that CD8^+^ T cells also express *LPAR5*, regulating respiration and cytotoxic activity in the tumor microenvironment [52,67]. Recent studies have demonstrated that fatty acid transport and oxidation play an essential role in tumor cell immune evasion [68]. CD36-mediated lipid accumulation regulated the differentiation of TAMs [69]. In macrophage and dendritic cells, FATP2 influenced the uptake of arachidonic acid, which regulated the synthesis of prostaglandin E2, resulting in an immune suppressive environment [70]. Moreover, in CD8 T cells, SLC3A2 and SLC7A11 regulated the lipid peroxidation and ferroptosis, influencing the efficacy of immune therapy. This study identifies that LPAR are predominantly expressed in macrophages and highlights the critical role of TAM-CD8^+^ T cell communication in the osteosarcoma microenvironment. AGPAT3 is shown to bridge LPA and PA metabolism with the tumor immune microenvironment, offering novel insights into their roles in osteosarcoma progression.

LPAR6, also known as P2RY5, is a G protein-coupled receptor. Previous reports have shown LPAR6 could regulate the activity of Gα12 and Gα13, and RhoA, affecting the function of T cells [71–73]. Given the important role of the LPA signaling in immune regulation, targeting this pathway could be a promising strategy for osteosarcoma immunotherapy. XAA has been reported as an LPAR6 inhibitor [34], but there is no further related clinical research. Therefore, in this study, we used virtual screening of FDA-approved drugs and identified 10 candidates that may bind to LPAR6. Among them, Dutasteride had the best docking score. These findings suggest that potential LPAR6 inhibitors can be identified from existing FDA-approved drugs, highlighting the therapeutic potential of targeting the LPA pathway.

AGPAT3 is an acyltransferase that converts LPA into PA, a process recognized by LPA receptors essential for B and T cell receptor signaling [74,75]. Our study demonstrates that AGPAT3 expression significantly correlates with signals transmitted from TAMs to CD8^+^ T cells. Furthermore, AGPAT3 knockdown in osteosarcoma cell lines, combined with co-culture experiments, confirmed that LPA regulates TAM-mediated secretion of IL-10 and IL-6 via the LPAR6 signaling pathway. Besides the enzymatic function, AGPAT3 was also involved in the regulation of Golgi morphology [76]. Our study primarily explored the function of AGPAT3 through LPA metabolism, while other functions of AGPAT3 in phospholipid metabolism may also influence membrane dynamics or vesicle trafficking, which warrants future exploration. In summary, low expression of *AGPAT3* in tumor cells facilitated the accumulation of LPA, which promoted the IL-6 and IL-10 expression of TAMs through LAPR signaling. The increased IL-6 and IL-10 in the microenvironment inhibited the function of T cells, inducing immune evasion in osteosarcoma. *AGPAT3* emerges as a critical gene with the lowest hazard ratio, positioning it as a promising therapeutic target in osteosarcoma. The development of AGPAT3-related agents deserves further study to explore their potential in enhancing the therapeutic effect of osteosarcoma.

This study demonstrated the correlation between AGPAT3 and osteosarcoma prognosis and identified the potential LAPR6 inhibitors. However, the mechanisms of LAPR6 regulating the ADGRE5-CD55 cell communication remain to be elucidated. The virtual screening identified FDA-approved Dutasteride as a potential inhibitor for LPAR6, but the detailed structural mechanisms and validation of its inhibitory function required further exploration. The cell line models and co-culture experiments could not fully simulate the efficiency *in vivo*. Moreover, the *in vivo* experiments are required to validate the function and off-target effects of LPAR6 inhibition. In addition, further pre-clinical experiments are needed to evaluate the safety and efficacy of LAPR6 inhibitors.

## 5 Conclusion

Our study highlights a strong correlation between glycerolipid metabolism and osteosarcoma prognosis, identifying AGPAT3 and ALDH7A1 as key genes associated with patient outcomes. Among them, AGPAT3 exhibits the lowest hazard ratio, underscoring its significance. AGPAT3 is closely linked to the regulation of the tumor immune microenvironment. Single-cell analysis further reveals that AGPAT3 regulates LPA levels, influencing TAM cytokine expression through the LPAR6 signaling pathway. These findings emphasize the critical role of AGPAT3-mediated phosphatidic acid metabolism in regulating the tumor immune microenvironment. AGPAT3 inhibition may offer a potential approach in combination with existing immunotherapies, pending further investigation.

## Supplementary Materials



## Data Availability

The data that support the findings of this study are available from the corresponding author upon reasonable request.

## Supplementary Materials

The supplementary material is available online at https://www.techscience.com/doi/10.32604/or.2025.070558/s1.
