# Supplementary material for: AGPAT3 Regulates Immune Microenvironment in Osteosarcoma via Lysophosphatidic Acid Metabolism
Source: Oncol Res. 2025 Dec 30;34(1):27. doi: 10.32604/or.2025.070558 (PMC12774546; doi:10.32604/or.2025.070558)
Supplement: Supplementary file 1 [file OncolRes-34-70558-s001.docx]

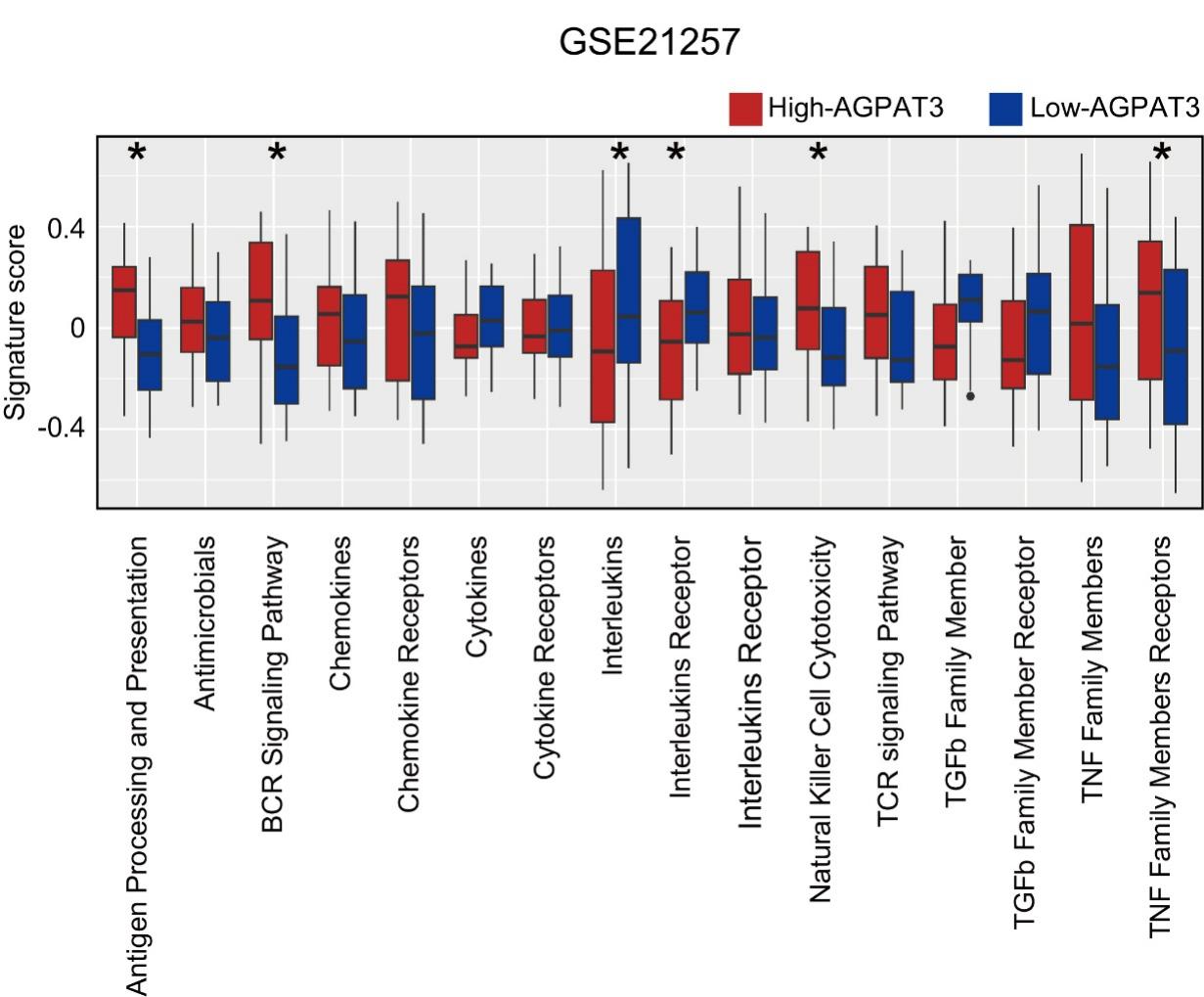


**Figure S1: Immune signature of GSE21257:** Signature scores and differential expression analysis of immune-related pathways in high- and low-*AGPAT3* expression groups. Data are presented as the mean ± SD. **p* < 0.05, by Student *t*-test.
